# Supplementary material for: Complex Admixture Preceded and Followed the Extinction of Wisent in the Wild
Source: Mol Biol Evol. 2016 Dec 21;34(3):598–612. doi: 10.1093/molbev/msw254 (PMC5356474; doi:10.1093/molbev/msw254)
Supplement: Supplementary Data [file msw254_Supp.pdf]

SUPPORTING INFORMATION FOR ONLINE PUBLICATION

Complex admixture preceded and followed the extinction of wisent in the wild

Karolina Węcek[1,2], Stefanie Hartmann[2], Johanna L. A. Paijmans[2], Ulrike Taron[2], Georgios Xenikoudakis[2], James A. Cahill[3], Peter D. Heintzman[3], Beth Shapiro[3,4], Gennady Baryshnikov[5], Aleksei N. Bunevich[6], Jennifer J. Crees[7], Roland Dobosz[8,9], Ninna Manaserian[10], Henryk Okarma[11], Małgorzata Tokarska[12], Samuel T. Turvey[7], Jan M. Wójcik[12], Waldemar Żyła[8], Jacek M. Szymura[1], Michael Hofreiter[2], Axel Barlow[2]

TABLE OF CONTENTS

Figure S1	A phylogeny of wisent and the Bovini, inferred from a partitioned maximum likelihood (ML) analysis of whole mitochondrial genomes…...	2
Figure S2	DNA fragmentation and deamination patterns of founding wisent, PLANTA, sequencing reads………………………………………………	3
Figure S3	DNA fragmentation and deamination patterns of founding wisent, PLATEN, sequencing reads………………………………………….....	4
Figure S4	DNA fragmentation and deamination patterns of Caucasian wisent, Cc1, sequencing reads.……………………………………………………..	5
Figure S5	DNA fragmentation and deamination patterns of Caucasian wisent, Cc2, sequencing reads……………………………………..……………….	6
Table S1	Species and accession data for sequences included in the mitochondrial genome phylogenetic analysis……………………………………………..	7
Table S2	Full details of sequencing and mapping results for modern wisent samples…………………………………………………………………….	9
Table S3	Full details of sequencing and mapping results for archival wisent samples…………………………………………………………………….	10
Table S4	Full details of mapping results for data downloaded from the NCBI Short Read Archive…………………………………………………………………	13
Table S5	Detailed *D* statistic values………………………………….……………...	17
Table S6	$\hat{f}$ results.………………………………………………………………	20

Figure S1. A phylogeny of wisent (A) and the Bovini (B), inferred from a partitioned maximum likelihood (ML) analysis of whole mitochondrial genomes. (A) is an expansion of the region in the blue box in (B). The green box in (A) highlights the two haplotypes found in sampled historic and modern individuals: one in Caucasian wisent (Cc2) and the other one (KY055664) in all remaining historical and modern wisent (see Table S1). Tips are coloured based on whether the haplotype occurs in modern individuals (black), or only in historical or ancient individuals, and therefore likely extinct (red). The outgroup, *Pseudoryx nghetinhensis*, is not shown. Branch support is indicated by bootstrap percentages based on 500 ML bootstrap replicates (above branches) and Bayesian posterior probabilities (below branches).

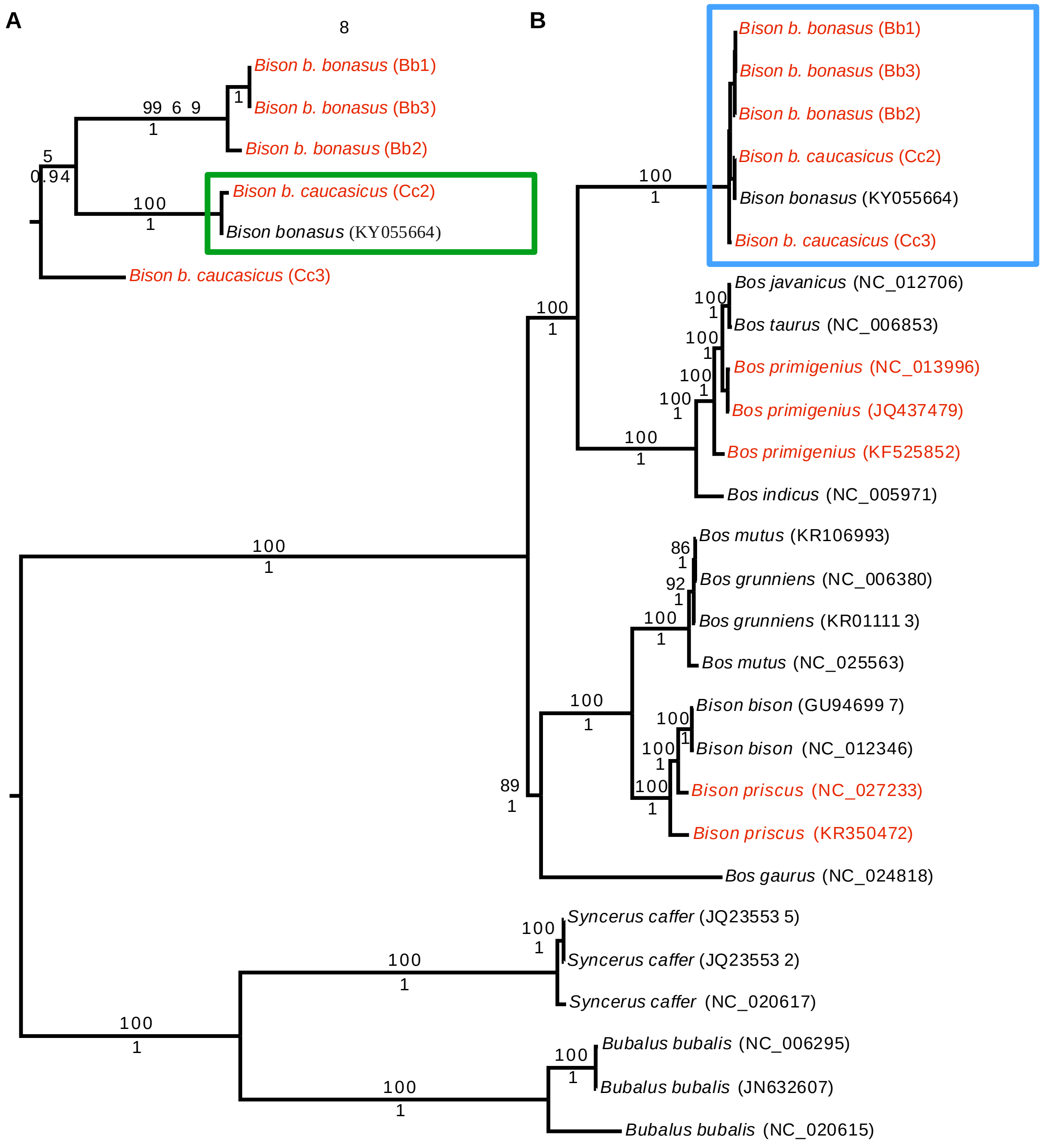

Figure S2. DNA fragmentation (upper four plots) and deamination (lower two plots) patterns of founding wisent, PLANTA, sequencing reads. For deamination plots, red lines show the frequency of C to T substitutions (Y axes) in the sequenced historical DNA fragments relative to the reference genome at the 5’ (left plot) and 3’ (right plot) fragment ends. X axes show sequenced positions moving internally from the 5’ (positive values) and 3’ (negative values) fragment ends. Elevated rate of C to T substitutions at fragment ends are indicative of DNA damage.

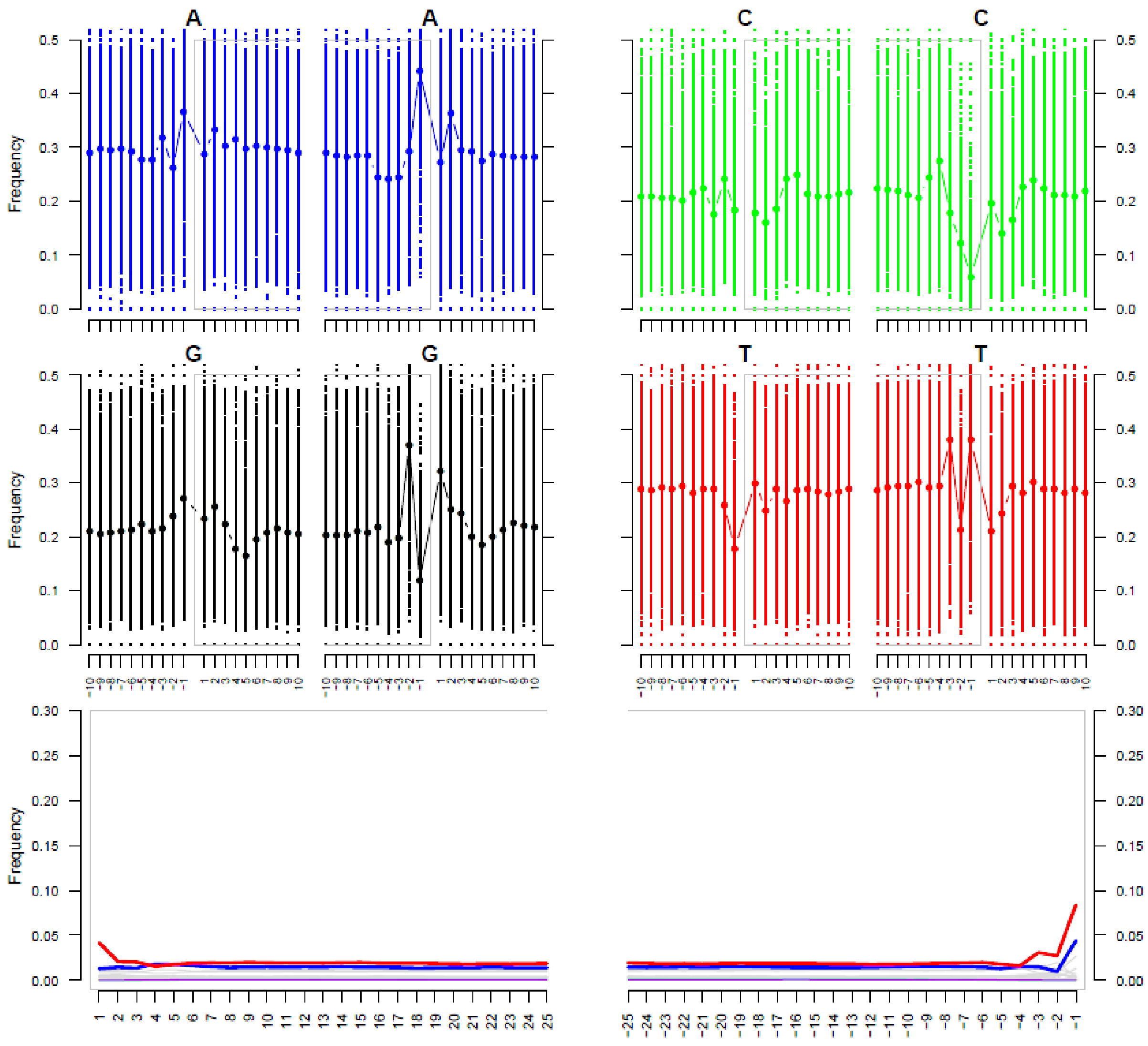

Figure S3. DNA fragmentation (upper four plots) and deamination (lower two plots) patterns of founding wisent, PLATEN, sequencing reads.

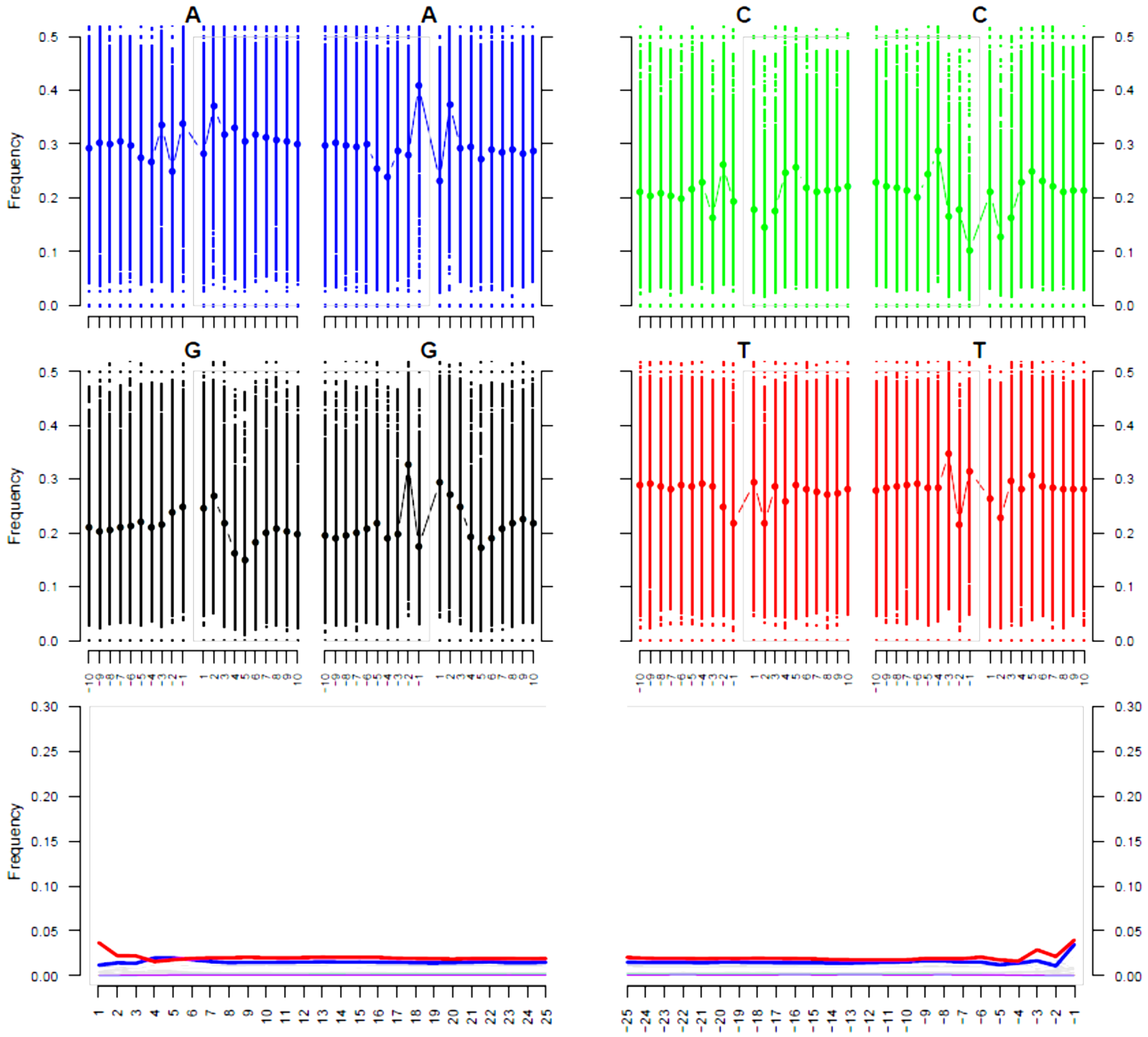

Figure S4. DNA fragmentation (upper four plots) and deamination (lower two plots) patterns of Caucasian wisent, Cc1, sequencing reads.

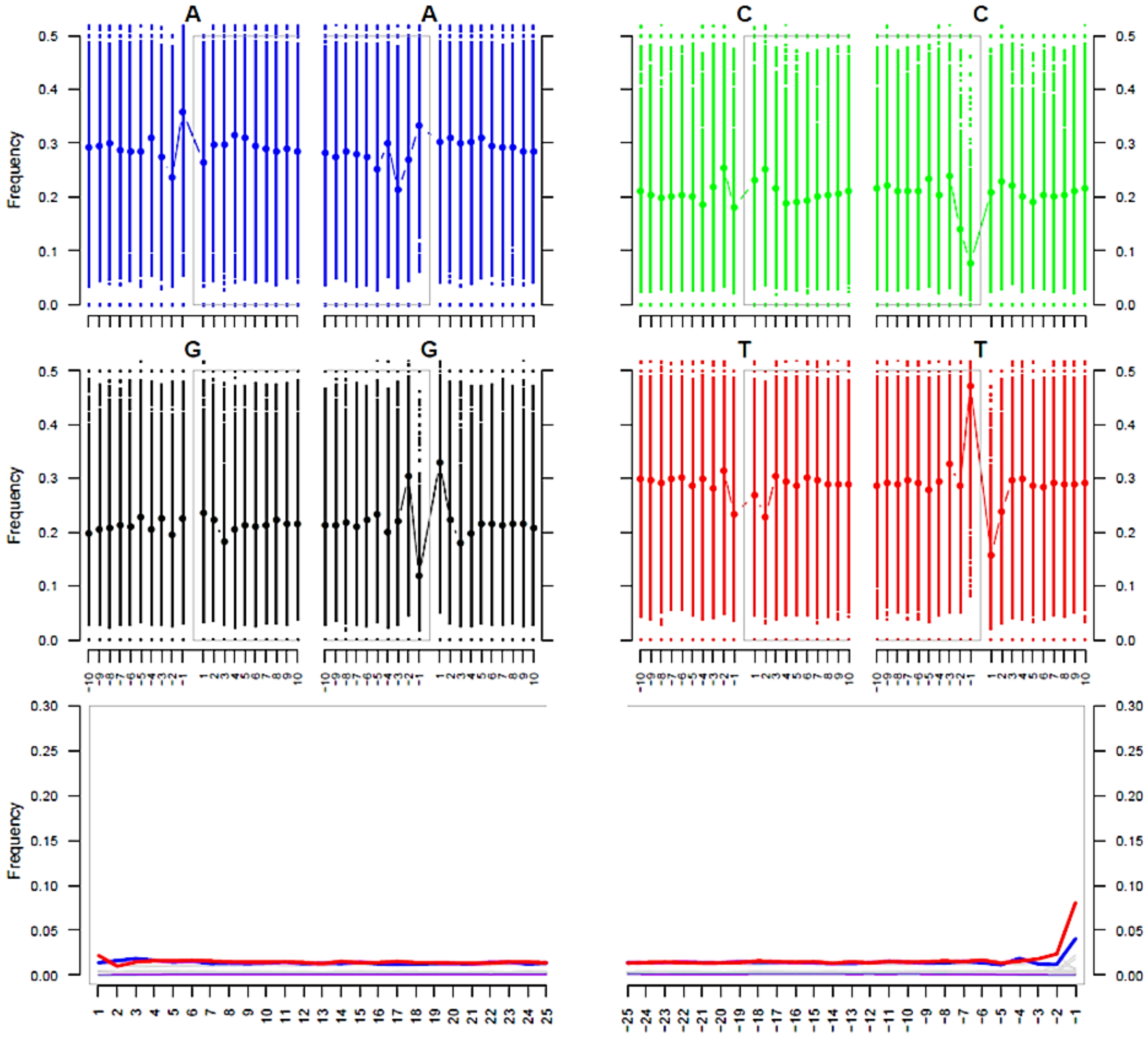

Figure S5. DNA fragmentation (upper four plots) and deamination (lower two plots) patterns of Caucasian wisent, Cc2, sequencing reads.

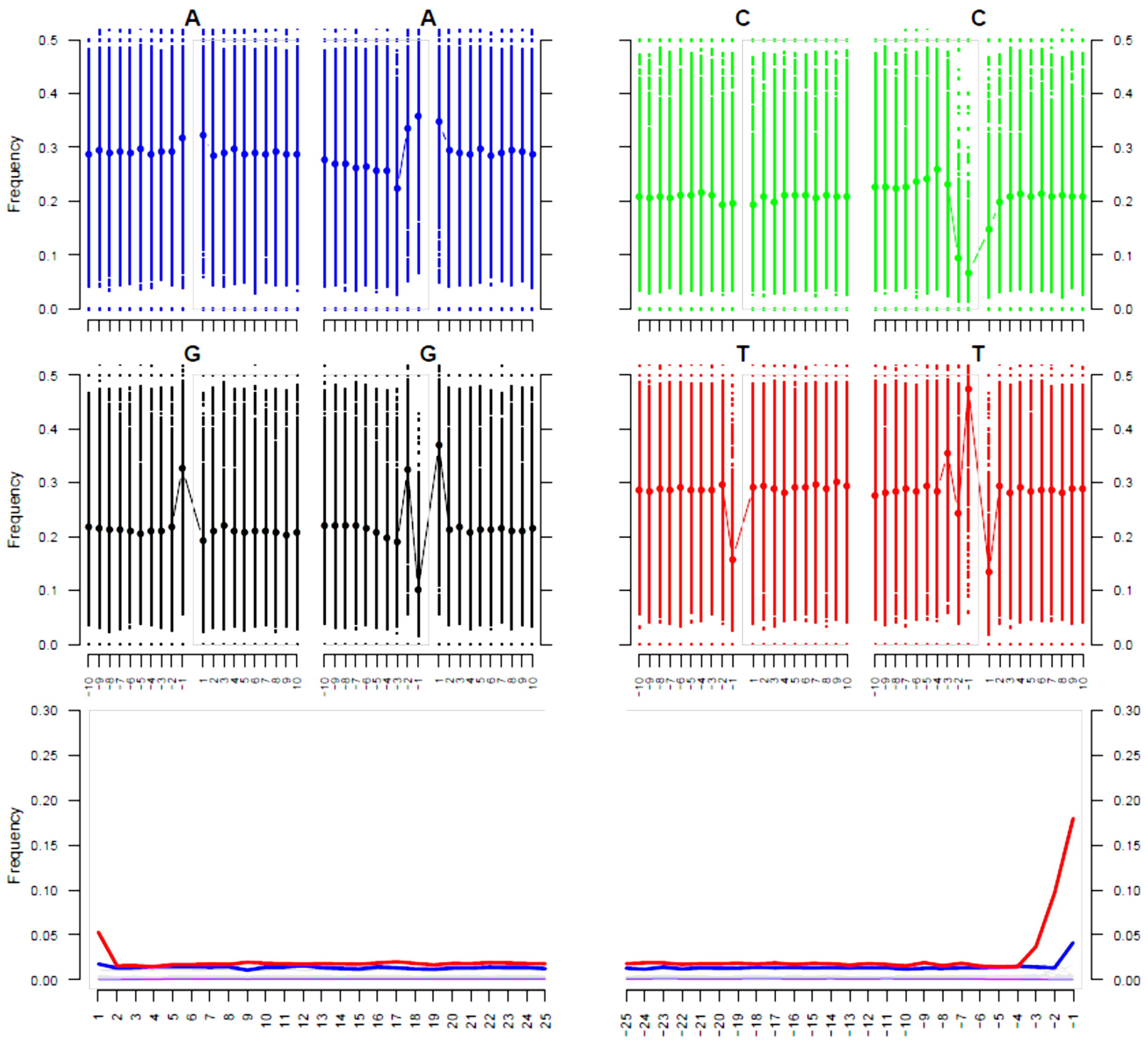

Table S1. Species and accession data for sequences included in the mitochondrial genome phylogenetic analysis.

Species		Status	Genbank accession	Location	Notes
scientific	common				
Bubalus bubalis	Water buffalo	Extant	NC_006295		
Bubalus bubalis	Water buffalo	Extant	JN632607		
Bubalus depressicornis	Lowland anoa	Extant	NC_020615		
Syncerus caffer	African buffalo	Extant	NC_020617		
Syncerus caffer	African buffalo	Extant	JQ235535		
Syncerus caffer	African buffalo	Extant	JQ235532		
Bison bison	American bison	Extant	NC_012346		
Bison bison	American bison	Extant	GU946997		
Bison priscus	Steppe bison	Extinct	NC_027233		
Bison priscus	Steppe bison	Extinct	KR350472		
Bison bonasus	Wisent/European bison – Lowland line	Extant	KY055664	Białowieża Forest, Polish part, Poland	MdL1; this study
Bison bonasus	Wisent/European bison – Lowland line	Extant	KY055664	Białowieża Forest, Polish part, Poland	MdL1a; this study
Bison bonasus	Wisent/European bison – Lowland line	Extant	KY055664	Białowieża Forest, Belarusian part, Belarus	MdL2; this study
Bison bonasus	Wisent/European bison – Lowland line	Extant	KY055664	Białowieża Forest, Belarusian part, Belarus	MdL2a; this study
Bison bonasus	Wisent/European bison – Lowland line	Extant	KY055664	Białowieża Forest, Belarusian part, Belarus	MdL2b; this study
Bison bonasus	Wisent/European bison – Lowland-Caucasian line	Extant	KY055664	Dydiowa, Bieszczady Mts, Poland	MdLC; this study
Bison bonasus	Wisent/European bison – Lowland-Caucasian line	Extant	KY055664	Lesko, Bieszczady Mts, Poland	MdLCa; this study
Bison bonasus	Wisent/European bison – Lowland-Caucasian line	Extant	KY055664	Maniów, Bieszczady Mts, Poland	MdLCb; this study
Bison bonasus	Wisent/European bison – Lowland-Caucasian line	Extant	KY055664	Sukowate, Bieszczady Mts, Poland	MdLCc; this study
Bison bonasus	Wisent/European bison – Lowland-Caucasian line	Extant	KY055664	Sukowate, Bieszczady Mts, Poland	MdLCd; this study

Bison bonasus	Lowland wisent	Extant	KY055664	Pszczyna, Poland	PLANTA; this study
Bison bonasus	Lowland wisent	Extant	KY055664	Pszczyna, Poland	PLATEN; this study
Bison b. bonasus	Lowland wisent	Extant	KX553930	Styria, Austria	Bb1; this study
Bison b. bonasus	Lowland wisent	Extant	KX553931	Styria, Austria	Bb2; this study
Bison b. bonasus	Lowland wisent	Extant	KX553932	Upper Austria, Austria	Bb3; this study
Bison b. caucasicus	Caucasian wisent	Extinct	KY055664	Kuban Oblast, Russia	Cc1; this study
Bison b. caucasicus	Caucasian wisent	Extinct	KX553933	North Ossetia-Alania, Russia	Cc2; this study
Bison b. caucasicus	Caucasian wisent	Extinct	KX553934	Sevan Lake region, Armenia	Cc3; this study
Bos grunniens	Yak (domesticated)	Extant	NC_006380.3		
Bos grunniens	Yak (domesticated)	Extant	KR011113		
Bos mutus	Yak (wild)	Extant	NC_025563		
Bos mutus	Yak (wild)	Extant	KR106993		
Bos primigenius	Aurochs	Extinct	NC_013996		
Bos primigenius	Aurochs	Extinct	JQ437479		
Bos primigenius	Aurochs	Extinct	KF525852		
Bos taurus	Domestic cattle	Extant	NC_006853		
Bos indicus	Zebu	Extant	NC_005971		
Bos gaurus	Gaur	Extant	NC_024818		
Bos javanicus	Banteng	Extant	NC_012706		
Pseudoryx nghetinhensis	Saola	Extant	NC_020616		Outgroup

All Genbank accessions are version one (.1) unless otherwise stated.

Table S2. Full details of sequencing and mapping results for modern wisent samples. SR – merged reads, PE – unmerged reads.

Taxon	Sample code	Sample name	Library type	Sequencing kit	Total read pairs	Merged and 30 bp+	Merged and 30bp+ /Total	Discarded	Discarded / Total	Unmerged reads (PE)	Total no. of reads used for mapping	BWA mismatch
Wisent (*Bison bonasus*)	MdL1	Z3	Double-stranded	High Output Kit, 75 bp PE	94456768	1673302	0.018	39034	0.0004	92744432	94417734	0.04
	MdL2	868			92027266	1605596	0.017	38499	0.0004	90383171	91988767	
	MdLC	Dy			83975270	1536928	0.018	33491	0.0004	82404851	83941779	

		Mapped to water buffalo nDNA (GenBank accession no. GCA_000471725.1)										
		SR	PE	Total	Uniq map to nucl ref	Uniq map / Total for map	Duplicates	Duplicates / Mapped SR	Duplicates / Mapped PE	Duplicates / Mapped Total	Total bp	Average coverage
Wisent (*Bison bonasus*)	MdL1	681232	59611336	60292568	59373802	0.629	15474	903292	918766	0.015	4521078762	1.59
	MdL2	692900	60862530	61555430	60608608	0.659	15507	931315	946822	0.015	4614409704	1.63
	MdLC	664763	55703290	56368053	55499836	0.661	13998	854219	868217	0.015	4227688598	1.49

		Mapped to zebu nDNA (GenBank accession no. GCA_000247795.2)										
		SR	PE	Total	Uniq map to nucl ref	Uniq map / Total for map	Duplicates	Duplicates / Mapped SR	Duplicates / Mapped PE	Duplicates / Mapped Total	Total bp	Average coverage
Wisent (*Bison bonasus*)	MdL1	918374	93693209	94611583	93037812	0.985	10526	1563245	1573771	0.017	7086721751	2.65
	MdL2	929613	95184458	96114071	94496465	1.027	10197	1607409	1617606	0.017	7196957491	2.69
	MdLC	899008	86595462	87494470	86021091	1.025	9673	1463706	1473379	0.017	6554700827	2.45

Table S3. Full details of sequencing and mapping results for archival wisent samples. SR – merged reads, PE – unmerged reads.

Taxon	Sample code	Sample name	Library type	Sequencing type	Sequencing kit	Total read pairs	Merged and 30 bp+	Merged and 30bp+ / Total	Discarded	Discarded / Total	Total reads used for mapping	BWA mismatch
Founders (*Bison b. bonasus*)	PLANTA	F42 PLANTA	Single-stranded	test	High Output Kit, 75 bp, PE	9123224	7445736	0.816	812463	0.089	7445736	0.04
				deep1	High Output Kit, 75 bp, SE	83685696	68554220	0.819	5489296	0.066	14224742	
				deep2	High Output Kit, 150 bp, PE	224815248	182038047	0.810	34706242	0.154	182038047	
				all data							203708525	
	PLATEN	M158 PLATEN		test	High Output Kit, 75 bp, PE	5474216	4313268	0.788	803445	0.147	4313268	
				deep1	High Output Kit, 75 bp, SE	26063084	22579039	0.866	3171599	0.122	22891485	
				deep2	High Output Kit, 150 bp, PE	227049648	175311357	0.772	45935293	0.202	175311357	
				all data							202516110	
Caucasian wisent (*Bison b. caucasicus*)	Cc1	8853		test	MiSeq Reagent Kit v3, 75 bp, PE	860901	722324	0.839	59002	0.069	722324	
				deep	High Output Kit, 75 bp, SE	381037631	283031189	0.743	23797618	0.062	357240013	
				all data							357962337	
	Cc2	22533		test	MiSeq Reagent Kit v3, 75 bp, PE	583050	504300	0.865	39800	0.068	504300	
				deep	High Output Kit, 75 bp, PE	330725680	266366670	0.805	27499199	0.083	266366670	
				all data							266870970	

Sample code	Sequencing type	Mapped to water buffalo nDNA (GenBank accession no. GCA_000471725.1)						
		SR	Uniq mapped to ref	Uniq mapped / Total for mapp	Duplicates	Duplicates / Mapped	Total bp	Average coverage
PLANTA	test	3404575	3333246	0.448	71329	0.021	205104971	0.072
	deep1	5463639	5389130	0.379	74509	0.014	313564372	0.111
	deep2	81971697	78669477	0.432	3302220	0.040	4959274186	1.749
	all data	70096827	67619347	0.332	2477480	0.035	4247491314	1.498
PLATEN	test	1746763	1708642	0.396	38121	0.022	95305368	0.034
	deep1	8146736	7921251	0.346	225485	0.028	423583261	0.149
	deep2	70325550	67092471	0.383	3233079	0.046	3751732087	1.323
	all data	74241817	70743685	0.349	3498132	0.047	3948880425	1.392
Cc1	test	135956	135740	0.188	216	0.002	9053505	0.003
	deep	56371128	53848774	0.151	2522354	0.045	3311112338	1.167
	all data	51737927	49548233	0.138	2189694	0.042	3047011245	1.074
Cc2	test	81496	81379	0.161	117	0.001	5312737	0.002
	deep	42136809	40490624	0.152	1646185	0.039	2620646703	0.924
	all data	42055313	40412164	0.151	1643149	0.039	2615503331	0.922

Sample code	Sequencing type	Mapped to zebu nDNA (GenBank accession no. GCA_000247795.2)						
		SR	Uniq mapped to ref	Uniq mapped / Total for mapping	Duplicates	Duplicates / Mapped	Total bp	Average coverage
PLANTA	test	4399394	4311321	0.579	88073	0.012	270221278	0.101
	deep1	7233018	7139867	0.502	93151	0.007	419837096	0.157
	deep2	106813785	102678676	0.564	4135109	0.023	6635984198	2.482
	all data	118446197	113628236	0.558	4817961	0.024	7299046803	2.730
PLATEN	test	2253290	2206347	0.512	46943	0.011	125792200	0.047
	deep1	10696173	10416147	0.455	280026	0.012	564643066	0.211
	deep2	91055261	87208248	0.497	3847013	0.022	5008178668	1.873
	all data	104004724	99240615	0.490	4764109	0.024	5669551099	2.120
Cc1	test	176546	176364	0.244	182	0.000	11960620	0.004
	deep	75065014	72086682	0.202	2978332	0.008	4470657635	1.672
	all data	75241560	72256423	0.202	2985137	0.008	4482222671	1.676
Cc2	test	108523	108402	0.215	121	0.000	7174444	0.003
	deep	56205819	54242773	0.204	1963046	0.007	3562372775	1.332
	all data	56314342	54347784	0.204	1966558	0.007	3569352473	1.335

Table S4. Full details of mapping results for data downloaded from the NCBI Short Read Archive. SR – merged reads, PE – unmerged reads.

**Analysis – mapped to**		**Species**		**SRA run number**	**Total read pairs**	**Merged and 30 bp+**	**Merged and 30bp+ / Total**	**Discarded**	**Discarded / Total**	**Unmerged reads (PE)**	**Total reads used for mapping**	**BWA mismatch**
**water buffalo**	**zebu**	**scientific**	**common**									
D statistic, $\hat{f}$	phylogeny	*Bos taurus*	domestic cattle	SRR934413	238847676	479705	0.002	120919	0.001	238247052	238726757	0.04
				SRR934414	230316320	464472	0.002	140107	0.000	229711741	230176213	
D statistic	phylogeny	*Bos primigenius*	aurochs	SRR2460709	1016735799	520683218	0	2468	0		1016733331	
	phylogeny	*Bubalus bubalis*	water buffalo	SRR032564	206417535	1496100	0.007	410002	1.99E-03	204511433	206007533	
				SRR034148	209373123	16980828	0.081	524472	2.50E-03	191867823	208848651	
				SRR034232	195014495	17184254	0.088	440197	2.26E-03	177390044	194574298	
				SRR035526	172356007	7550202	0.044	50269837	2.92E-01	114535968	122086170	
				SRR060735	169923020	114770	0.001	114770	6.75E-04	169693480	169808250	
				SRR0_32564+…+_60735							207504227	
density plots	phylogeny	*Bos grunniens*	yak	SRR2059884	120740246	50493752	0.418	130340	1.08E-03	70116154	120609906	
				SRR1047220	96708130	755974	0.008	2095	2.17E-05	95950061	96706035	
				SRR1047221	98661866	659076	0.007	2015	2.04E-05	98000775	98659851	
				SRR962824	92733380	707858	0.008	2648	2.86E-05	92022874	92730732	
				SRR962825	99974841	929587	0.009	4159	4.16E-05	99041095	99970682	
				SRR962826	91283932	1099526	0.012	3984	4.36E-05	90180422	91279948	
				SRR962827	89026186	787360	0.009	2949	3.31E-05	88235877	89023237	
density plots	phylogeny	*Bos taurus*	domestic cattle	DRR001769	113132803	32880649	0.291	1207203	1.07E-02	79044951	111925600	
				SRR1525627	210668440	16504311	0.078	43450	2.06E-04	194120679	210624990	
				SRR567217	117029482	476258	0.004	7032	6.01E-05	116546192	117022450	
				SRR567218	195413620	720391	0.004	11257	5.76E-05	194681972	195402363	
				SRR5672_17+_18							123446310	

				SRR934420	216161654	1209329	0.006	21176	9.80E-05	214931149	216140478
				SRR1805809	163405319	578830	0.004	24765	1.52E-04	162801724	163380554
				DRR000426	75320405	7450315	0.099	277520	3.68E-03	67592570	75042885
				DRR000427	92855805	7280650	0.078	319499	3.44E-03	85255656	92536306
				DRR000428	76946227	6804769	0.088	226487	2.94E-03	69914971	76719740
				DRR000429	104954914	8406579	0.080	323924	3.09E-03	96224411	104630990
				DRR0004_26+…+_29							201326065
				SRR567184	251331631	1087090	0.004	11489	4.57E-05	250233052	251320142
				SRR567198	95548138	428639	0.004	4369	4.57E-05	95115130	95543769
				SRR5671_84+_98							142041257

SRA run number	Mapped to water buffalo nDNA (GenBank accession no. GCA_000471725.1)										
	SR	PE	Total	Uniq mapped to nucl ref	Uniq mapped / Total for mapp	Duplicates			Duplicates / Mapped	Total bp	Average coverage
						SR	PE	Total			
SRR934413	77465	180997747	181075212	143848243	0.603	13273	37213696	37226969	0.206	14506214554	5.115
SRR934414	74330	184634778	184709108	158079423	0.687	13709	26615976	26629685	0.144	15932546511	5.618
SRR2460709	89731327			81326176	0.080	8405151			0.094	3645868657	1.285
SRR032564											
SRR034148											
SRR034232											
SRR035526											
SRR060735											
SRR0_32564+…+_60735											
SRR2059884	23883248	54187644	78070892	76928870	0.638	549741	592281	1142022	0.015	8535503292	3.010
SRR1047220	126918	64714610	64841528	63556931	0.657	16029	1268568	1284597	0.020	5711720183	2.014
SRR1047221	29477	66526681	66556158	64030835	0.649	22781	2502542	2525323	0.038	5745455083	2.026
SRR962824	101469	62335158	62436627	54774145	0.591	22454	7640028	7662482	0.123	4908615326	1.731
SRR962825	117847	61239580	61357427	58322049	0.583	18075	3017303	3035378	0.049	5223670340	1.842
SRR962826	245957	58464212	58710169	54184661	0.594	20811	4504697	4525508	0.077	4859233429	1.713
SRR962827	137423	59846752	59984175	54603237	0.613	14875	5366063	5380938	0.090	4898134845	1.727
DRR001769	11440674	37680226	49120900	48266189	0.431	516838	337873	854711	0.017	5198838131	1.833
SRR1525627	7294154	135177669	142471823	140173343	0.666	167047	2131433	2298480	0.016	14378571610	5.070
SRR567217	34270	47153211	47187481	46778055	0.400	11112	398314	409426	0.009	3444552509	1.215
SRR567218	51047	77605295	77656342	76668255	0.392	16875	971212	988087	0.013	5606859126	1.977
SRR5672_17+_18			123446310	122510971	0.992			935339	0.008	8854024023	3.122
SRR934420	411729	166992353	167404082	165960267	0.768	17767	1426048	1443815	0.009	16743811943	5.904
SRR1805809	71298	90404542	90475840	90136790	0.552	7625	331425	339050	0.004	9010122832	3.177
DRR000426	2923372	39686824	42610196	42304570	0.564	71918	233708	305626	0.007	3242386653	1.143
DRR000427	2927630	50176507	53104137	52441480	0.567	88670	573987	662657	0.012	4017269546	1.416

DRR000428	2773518	42879645	45653163	45386147	0.592	62659	204357	267016	0.006	3479251499	1.227
DRR000429	3480388	58141011	61621399	61193868	0.585	90641	336890	427531	0.007	4689183604	1.653
DRR0004_26+…+_29			201326065	199422800	0.991			1903265	0.009	15238028624	5.373
SRR567184	68357	103065941	103134298	2259890	0.009	20499	853909	874408	0.008	7637589717	2.693
SRR567198	27486	39900959	39928445	39781367	0.416	7227	139851	147078	0.004	2980254500	1.051
SRR5671_84+_98			142041257	141455657	0.996			585600	0.004	10543641683	3.718

SRA run number	Mapped to zebu nDNA (GenBank accession no. GCA_000247795.2)										
	SR	PE	Total	Uniq mapped to nucl ref	Uniq mapped / Total for mapp	Duplicates			Duplicates / Mapped	Total bp	Average coverage
						SR	PE	Total			
SRR934414	87745	151133612	151221357	266142638	1.156	3596	24651143	24654739	0.163	26866401151	10.05
SRR2460709	115897909			104281079	0.103			11616830	0.100	4676788198	1.75
SRR032564	690009	6136348	6826357	6801729	0.996	10058	14570	24628	0.004	523399599	0.20
SRR034148	7254959	75698327	82953286	79637860	0.960	358054	2957372	3315426	0.040	6237121023	2.33
SRR034232	7415111	74837858	82252969	79388246	0.965	340717	2524006	2864723	0.035	6220532766	2.33
SRR035526	362230	28384360	28746590	13272267	0.462	356555	15117768	15474323	0.538	477327387	0.18
SRR060735	17354	87526774	87544128	28404125	0.324	4989	59135014	59140003	0.676	1021455908	0.38
SRR0_32564+…+_60735				202618910	0.976			4885317	0.024	14107118827	5.28

Table S5. Detailed *D* statistic results. In red significant results are shown. Results highlighted in grey are presented also in Figure 5.

comparison	*D* statistic value	Jackknife test σ value	*Z* score
**COMPARISONS WITHIN MODERN WISENT (*B. BONASUS*) AND FOUNDERS (*B. B. BONASUS*)**			
Among modern individuals			
MdL2_MdL1_MdLC	0.060	0.028	2.162
MdL1_MdLC_MdL2	-0.237	0.026	9.270
MdL2_MdLC_MdL1	-0.180	0.027	6.592
Contribution of founders to modern			
MdL1_MdL2_PLANTA	0.001	0.026	0.040
MdL1_MdL2_PLATEN	-0.017	0.028	0.608
MdL1_MdLC_PLANTA	-0.153	0.025	6.140
MdL1_MdLC_PLATEN	-0.177	0.026	6.867
MdL2_MdLC_PLANTA	-0.145	0.024	5.947
MdL2_MdLC_PLATEN	-0.153	0.027	5.679
As above but with different order. Elevated D values basically support more recent ancestry of L and LC than L and founders			
PLANTA_PLATEN_MdL1	0.012	0.021	0.559
PLANTA_PLATEN_MdL2	-0.010	0.020	0.514
PLANTA_PLATEN_MdLC	-0.016	0.020	0.803
**ADMIXTURE WITH CAUCASIAN WISENT (*B. B. CAUCASICUS*)**			
Caucasian wisent and founders			
PLANTA_PLATEN_Cc1	-0.039	0.014	2.774
PLANTA_PLATEN_Cc2	-0.047	0.013	3.606
Caucasian admixture with modern individuals, relative to founders			
MdL1_PLANTA_Cc1	-0.138	0.018	7.835
MdL1_PLANTA_Cc2	-0.137	0.016	8.810
MdL1_PLATEN_Cc1	-0.173	0.018	9.624
MdL1_PLATEN_Cc2	-0.175	0.016	10.855
MdL2_PLANTA_Cc1	-0.152	0.017	8.693
MdL2_PLANTA_Cc2	-0.135	0.015	8.296
MdL2_PLATEN_Cc1	-0.190	0.017	10.920
MdL2_PLATEN_Cc2	-0.177	0.016	11.396
MdLC_PLANTA_Cc1	-0.241	0.019	12.860
MdLC_PLANTA_Cc2	-0.149	0.016	9.360
MdLC_PLATEN_Cc1	-0.266	0.019	14.328
MdLC_PLATEN_Cc2	-0.187	0.015	12.164
Caucasian wisent and modern individuals			
MdL1_MdL2_Cc1	0.022	0.021	1.012
MdL1_MdL2_Cc2	0.001	0.019	0.058

MdL1_MdLC_Cc1	0.143	0.022	6.418
MdL1_MdLC_Cc2	0.028	0.019	1.492
MdL2_MdLC_Cc1	0.117	0.023	5.078
MdL2_MdLC_Cc2	0.029	0.018	1.598
Differential admixture between modern and founding individuals and the two Caucasian individuals			
Cc1_Cc2_MdL1	-0.038	0.015	2.565
Cc1_Cc2_MdL2	-0.048	0.014	3.336
Cc1_Cc2_MdLC	-0.106	0.016	6.780
Cc1_Cc2_PLANTA	-0.037	0.014	2.654
Cc1_Cc2_PLATEN	-0.038	0.014	2.663
**ADMIXTURE WITH DOMESTIC CATTLE (*BOS TAURUS*)**			
Domestic cattle or aurochs admixture?			
DC1_aurochs_MdL1	-0.413	0.009	44.039
DC2_aurochs_MdL1	-0.412	0.009	46.072
DC1_aurochs_MdL2	-0.409	0.009	45.040
DC2_aurochs_MdL2	-0.415	0.009	47.257
DC1_aurochs_MdLC	-0.408	0.009	43.833
DC2_aurochs_MdLC	-0.413	0.009	45.159
DC1_aurochs_PLANTA	-0.348	0.009	36.878
DC2_aurochs_PLANTA	-0.351	0.009	38.491
DC1_aurochs_PLATEN	-0.315	0.010	31.826
DC2_aurochs_PLATEN	-0.319	0.010	32.677
DC1_aurochs_Cc1	-0.325	0.010	32.089
DC2_aurochs_Cc1	-0.323	0.010	31.945
DC1_aurochs_Cc2	-0.345	0.010	34.145
DC2_aurochs_Cc2	-0.346	0.010	34.618
MdL1_DC1_aurochs	0.949	0.001	733.910
MdL1_DC2_aurochs	0.949	0.001	737.934
MdL2_DC1_aurochs	0.948	0.001	739.371
MdL2_DC2_aurochs	0.949	0.001	744.363
MdLC_DC1_aurochs	0.948	0.001	725.067
MdLC_DC2_aurochs	0.949	0.001	726.495
PLANTA_DC1_aurochs	0.948	0.001	738.668
PLANTA_DC2_aurochs	0.948	0.001	750.189
PLATEN_DC1_aurochs	0.948	0.001	730.198
PLATEN_DC2_aurochs	0.949	0.001	734.874
Cc1_DC1_aurochs	0.950	0.001	748.172
Cc1_DC2_aurochs	0.950	0.001	746.504
Cc2_DC1_aurochs	0.952	0.001	798.197
Cc2_DC2_aurochs	0.952	0.001	796.582
Differential domestic cattle admixture among founders and historical Caucasian			
PLANTA_Cc1_DC1	-0.050	0.009	5.571
PLANTA_Cc2_DC1	-0.051	0.009	5.611
PLATEN_Cc1_DC1	0.013	0.009	1.338
PLATEN_Cc2_DC1	0.005	0.010	0.487

PLANTA_PLATEN_DC1	-0.078	0.011	7.288
Cc2_Cc1_DC1	0.007	0.010	0.632
Cc1_PLANTA_DC1	0.044	0.009	4.836
Differential domestic cattle admixture in modern wisent relative to founders and Caucasian			
MdL1_PLANTA_DC1	-0.309	0.011	28.336
MdL1_PLATEN_DC1	-0.367	0.011	33.115
MdL2_PLANTA_DC1	-0.301	0.011	26.716
MdL2_PLATEN_DC1	-0.367	0.011	32.310
MdLC_PLANTA_DC1	-0.277	0.011	25.203
MdLC_PLATEN_DC1	-0.329	0.011	29.070
MdL1_Cc1_DC1	-0.244	0.010	25.041
MdL1_Cc2_DC1	-0.259	0.010	26.101
MdL2_Cc1_DC1	-0.244	0.010	25.518
MdL2_Cc2_DC1	-0.252	0.010	25.615
MdLC_Cc1_DC1	-0.245	0.010	24.139
MdLC_Cc2_DC1	-0.257	0.010	25.006
Differential domestic cattle admixture among modern wisent			
MdL1_MdL2_DC1	0.000	0.013	0.012
MdL1_MdLC_DC1	-0.004	0.013	0.335
MdL2_MdLC_DC1	-0.011	0.012	0.903
**ADMIXTURE WITH AUROCHS (*BOS PRIMIGENIUS*)**			
Differential aurochs admixture among founders and historical Caucasian			
PLANTA_Cc1_aurochs	-0.016	0.011	1.429
PLANTA_Cc2_aurochs	-0.051	0.012	4.421
PLATEN_Cc1_aurochs	0.018	0.012	1.551
PLATEN_Cc2_aurochs	-0.017	0.013	1.398
PLANTA_PLATEN_aurochs	-0.051	0.014	3.641
Cc2_Cc1_aurochs	0.044	0.013	3.366
Differential aurochs admixture in modern wisent relative to founders and Caucasian			
MdL1_PLANTA_aurochs	-0.130	0.013	9.640
MdL1_PLATEN_aurochs	-0.178	0.014	12.448
MdL2_PLANTA_aurochs	-0.121	0.013	9.185
MdL2_PLATEN_aurochs	-0.165	0.014	11.700
MdLC_PLANTA_aurochs	-0.112	0.014	8.236
MdLC_PLATEN_aurochs	-0.147	0.014	10.490
MdL1_Cc1_aurochs	-0.101	0.012	8.306
MdL1_Cc2_aurochs	-0.142	0.012	11.349
MdL2_Cc1_aurochs	-0.102	0.012	8.795
MdL2_Cc2_aurochs	-0.137	0.012	10.998
MdLC_Cc1_aurochs	-0.095	0.013	7.543
MdLC_Cc2_aurochs	-0.145	0.013	11.390

Table S6. $\hat{f}$ results. In the last column percentage of the genome resulted from hybridisation is given.

**comparison**	$\hat{f}$ **value**	**%**
Cc1_MdL1_DC1_DC2_buffalo	0.017	1.70
Cc1_MdL2_DC1_DC2_buffalo	0.017	1.69
Cc1_MdLC_DC1_DC2_buffalo	0.016	1.63
Cc2_MdL1_DC1_DC2_buffalo	0.032	3.24
Cc2_MdL2_DC1_DC2_buffalo	0.032	3.21
Cc2_MdLC_DC1_DC2_buffalo	0.032	3.19
PLATEN_MdL1_DC1_DC2_buffalo	0.030	3.02
PLATEN_MdL2_DC1_DC2_buffalo	0.030	3.03
PLATEN_MdLC_DC1_DC2_buffalo	0.030	3.04
PLANTA_MdL1_DC1_DC2_buffalo	0.024	2.44
PLANTA_MdL2_DC1_DC2_buffalo	0.024	2.44
PLANTA_MdLC_DC1_DC2_buffalo	0.025	2.45
